# Emotional Support, Depressive Symptoms, and Age-Related Alterations in Male Body Composition: Cross-Sectional Findings from the Men's Health 40+ Study

**DOI:** 10.3389/fpsyg.2017.01075

**Published:** 2017-06-29

**Authors:** Andreas Walther, Michel Philipp, Niclà Lozza, Ulrike Ehlert

**Affiliations:** ^1^Clinical Psychology and Psychotherapy, University of ZurichZurich, Switzerland; ^2^University Research Priority Program—Dynamics of Healthy Aging, University of ZurichZurich, Switzerland; ^3^Psychological Methods, Evaluation and Statistics, University of ZurichZurich, Switzerland

**Keywords:** depression, emotional support, body composition, sarcopenia, bioelectrical impedance analysis, men, psychosocial resilience factors

## Abstract

More depressive symptoms and low emotional support have been related to worse body composition. Body composition significantly deteriorates in aging men. Therefore, we aimed to examine whether high emotional support and low depressive symptoms are associated with better body composition and a decelerated age-related deterioration of body composition in aging men. A cross-sectional analysis including 269 self-reporting healthy men aged between 40 and 75 years living in the German-speaking part of Switzerland was conducted. Participants completed questionnaires on emotional support and depressive symptoms. The depression screening instrument was used to form a group with low (*N* = 225) and moderate (*N* = 44) depressive symptoms. Body mass index (BMI) and waist-to-hip ratio (WHR) were measured, and cell proportion (CP), fat mass (FM), and water balance (WB) were obtained using bioelectrical impedance analysis. Age-related associations emerged for WHR, CP, FM, and WB, but not for BMI. Emotional support was negatively associated with BMI, WHR, and WB, and only trend-wise with CP and FM. Group comparisons revealed that more depressive symptoms were associated with lower levels of CP and higher levels of WB. Both emotional support and depressive symptoms were significant moderators of the association between age and specific measures of body composition such as CP, FM, and WB. However, after correction for multiple testing for moderation analyses only the moderation effects of depressive symptoms on the association between age and WB and CP remained significant. Low depressive symptoms were associated with a better body composition as well as a decelerated decline in body composition and the role of emotional support acting as a buffer against age-related deterioration of body composition merits further investigation.

## Introduction

Emotional support is considered to be a powerful protective agent not only against depression but also against obesity (Vogelzangs et al., 2007; Sharpley et al., 2015). However, research on the relationship between emotional support and body composition is scarce and only unstandardized screening questions used as proxys for emotional support were used. In a study examining 2,917 elderly persons, inadequate emotional support (a main component of social support along with instrumental support) was positively associated with abdominal obesity, defined as a high waist circumference (Vogelzangs et al., 2007). However, inadequate emotional support was assessed via a single item asking “Could you have used more emotional support than received during the last year?” Participants, who indicated “some more” or “a lot more” were defined as having had inadequate emotional support. A longitudinal study following children from birth to the age of 26 years found that social isolation during childhood was associated with overweight, elevated cholesterol, and low high-density lipoprotein level (Caspi et al., 2006). This study operationalized childhood social isolation by repeated parent report on two items measuring peer problems (“tends to do things on his/her own; is rather solitary” and “not much liked by other children”). A third study used a single item to assess fathers' emotional support “Is there someone you can turn to for day-to-day emotional help with parenthood/raising children?” with the response options “yes” and “no” and subsequently measured overweight or obesity status of the children (Watt et al., 2012). In English-speaking Hispanic fathers the odds to have an overweight/obese child were significantly higher than in Whites, but reporting to have emotional support reduced the odds by 80%. These studies failed to measure emotional support with validated scales of emotional support and are therefore not unambiguously interpretable with regard to the construct of emotional support. Social isolation might be a proxy also for social support in general or for instrumental support and not only emotional support, while the question if one can turn to somebody for day-to-day emotional help with parenthood does not exclude a more general interpretation of social support also potentially including instrumental/informational support. Therefore, there is a need for studies investigating the relationship between body composition and emotional and other forms of social support with validated scales allowing to disentangle specific effects of emotional or instrumental support.

In contrast to the sparse findings regarding emotional support, the association between depressive symptoms and body composition has been proclaimed a major health care interest, as a dramatic increase in depression has been recorded worldwide (Bauer et al., 2013). An analysis of a large cross-sectional survey comprising 177,047 adults revealed a U-shaped association between depressive symptoms and BMI, and indicated gender differences (Zhao et al., 2009). Likewise, another study reported a U-shaped association between depressive symptoms and BMI in young men (Kim et al., 2015). Although this U-shaped association in young men is very consistent, it was reported to diminish in older men (McCrea et al., 2011). By contrast, a recent study examining 3,412 men aged between 50 and 102 years also found a U-shaped association between depressive symptoms and BMI, indicating that both underweight and obesity are associated with depressive symptoms in aging men (Noh et al., 2015). However, the association between depressive symptoms and BMI in self-reporting healthy men has not yet been investigated. Since, BMI is seen as a proxy of general health, no association might result when investigating only men with overall good health and only more sensitive measures might detect any differences in such a homogeneous group.

In the last decade, researchers have criticized the use of the BMI, and advocate measuring different aspects of body composition instead—ideally using bioelectrical impedance analysis (Talluri et al., 2003). However, literature on features of body composition and emotional support or depressive symptoms is scarce. A recent study showed that a reduction in depressive symptoms in 52 chronic obstructive pulmonary disease patients was accompanied by a reduction of FM as well as BMI (Catalfo et al., 2016). In the Health, Aging, and Body Composition Study, baseline depressive symptoms were found to result in an increase in abdominal obesity at 5-year follow-up, as measured by computed tomography scanning (Vogelzangs et al., 2008). Another recent study by Kahl et al. (2017) reported reduced muscle mass in 28 depressed men compared to 10 healthy controls indicating a male specific biological signature for depressive disorders as suggest earlier (Walther et al., 2017b). Finally, a large-scale study encompassing 4,511 participants aged from 35 to 69 reported strong positive associations between depressive symptoms and FM, as measured with a bioelectrical impedance analysis (Yu et al., 2014).

In addition, at around the age of 40 years, male body composition begins to undergo significant changes, which increase frailty. Lifetime risk of a fracture at the age of 45 has been reported to lie at 24% (Kanis et al., 2000). The age-related decrease in muscle mass as well as the age-related increase in fat mass plays a key role in frailty, indicating that deceleration of the age-related deterioration of body composition is an important prevention strategy against falls and fractures in older males. While BMI, WHR, FM, extracellular mass (ECM), extracellular water (ECW), and the WB show a continuous age-related increase, for body cell mass (BCM), fat free mass (FFM), or total body water (TBW), an age-related decline is consistently reported after the age of 40 (Kyle et al., 2001; World Health Organization, 2008b; Yamada et al., 2010). Multifactorial prevention and treatment strategies against age-related deterioration of body composition include physical exercise, nutrition control, and hormonal supplementation, but neglect to incorporate psychosocial risk and resilience factors (Campbell and Robertson, 2006). Given the previous literature reporting associations between emotional support, depressive symptoms, and body composition, the identification of potential modifying effects of these psychosocial factors on age-related deterioration of body composition emerges as promising health care goal.

This study pursued two objectives. The first objective was to examine associations of emotional support and depressive symptoms with body composition in self-reporting healthy men aged between 40 and 75 years. A second objective was to identify moderating effects of emotional support and depressive symptoms on the age-related deterioration of body composition in self-reporting healthy men aged between 40 and 75 years.

## Materials and methods

### Participants and procedure

This study is part of a research project on healthy aging in men—the Men's Health 40+ study, which has been previously described elsewhere (Walther et al., 2016). The sample of the present study consisted of 269 self-reporting healthy men aged 40–75 years recruited via web pages and flyers from the German speaking part of Switzerland. The health condition was controlled by the first question of the Short Form-36 (Brazier et al., 1992), which asked participants how they would describe their current health condition with response options “very bad,” “bad,” “fair,” “good,” and “very good.” Participants indicated to perceive their current health condition as either “very good,” “good,” or “fair.” Participants provided psychometric data on their depression status and social support as well as measures of body composition. Participants completed independently the psychometric questionnaires via a computer with Internet access. After completion of the questionnaires participants were invited to a biological examination at the Psychological Institute of the University of Zurich, where the bioelectrical impedance analysis was performed. Participants were prior informed to arrive at the laboratory at 7:45 a.m. after overnight fasting. All participants provided written informed consent to take part in the study. The local Ethics Committee of the Faculty of Arts at the University of Zurich approved the study protocol before data collection.

### Psychometric measures

The first section of the Berlin Social Support Scales (BSSS) was used to assess participants' perceived emotional (BSSS-ES), instrumental (BSSS-IS) and total social support (BSSS; Schulz and Schwarzer, 2003). We chose the BSSS to measure social and emotional support, as previous literature has highlighted the multidimensional approach as a strength of the instrument, with the use of only 17 items and being well-validated in German constituting main advantages (Wirtz et al., 2006). Participants were asked to rate whether they agree with statements such as “There are some people who truly like me” on a 4-point scale ranging from 1 (strongly disagree) to 4 (strongly agree). A sum score up to 68 can be achieved, with higher scores indicating higher perceived social support. For emotional support mean scores were calculated ranging from 1 to 4 with increments of 0.25. Cronbach's α, a measure for the internal consistency of a psychometric scale, ranges between 0.83 and 0.86 for the three BSSS scales, which is considered a good internal consistency.

The General Depression Scale—Long Version 2 (ADS-L2) was used to assess participants' depressive symptoms (Hautzinger et al., 2012). This German-language scale is based on the Center for Epidemiological Studies Depression Scale (CES-D), a self-report depression scale for research in the general population (Radloff, 1977), and was thus well-suited for this non-clinical sample. Moreover, a previous study used the CES-D scale to examine associations between depressive symptoms and abdominal obesity in a community setting of 30 to 64-year-old African American men, providing us with a point of comparison for the influence of depression scores on measures of obesity between these two samples (Cooper et al., 2013). A score of 16 or higher is considered clinically relevant (Radloff, 1977; Cooper et al., 2013). Cronbach's α for the ADS-L2 in our study was 0.82, thus demonstrating satisfactory internal consistency.

### Body composition

Weight, height, BMI, and WHR were measured using a Seca balance and stadiometer according to standard procedures described elsewhere (World Health Organization, 2008a). A bioelectrical impedance analysis (BIACORPUS RX 4000; BodyComp V 8.5; idiag AG, Switzerland) was used to assess participants' body composition. The bioelectrical impedance analysis was performed after overnight fasting with the participant in a lying position attaching two electrodes on each hand and foot with the required minimal distance between them. Obtained measures were: CP (%), BCM (kg), FFM (%/kg), FM (%/kg), ECM (%/kg), ECW (%/l), TBW (l), WB, and ECM-BCM ratio. For a further description of parameters, see Supplementary Material or (Kotler et al., 1996).

### Potential covariates and confounders

A set of covariates was used based on previous studies conducted in this area (Yu et al., 2014). Additional covariates were either health-related or body composition-related. Covariates included were age, marital status, smoking status and alcohol consumption, medication intake, drug consumption, education, and income, having had a cold or other illness during the last 2 weeks, nutrition style, and self-reported increase in intake of fatty or sweet food during the last 3 months. The current health condition, subjective effort to be healthy and the amount of hours of physical activity per week were further included as covariates. For “No/Yes” questions (e.g., “Are you taking any medication?”), covariates were coded as either “0” or “1.” For questions with more response options (e.g., “How would you describe your nutrition style?” with response options “very unhealthy,” “unhealthy,” “neither healthy nor unhealthy,” “healthy,” or “very healthy”), responses were coded from “0” to “4.” Therefore, covariates were used as continuous variables. For more detailed description of covariates see Supplementary Material.

### Statistical analysis

In a first step, associations between age, emotional support and different measures of body composition were analyzed using Spearman bivariate correlations (*r*_*s*_). The distribution of the BSSS-ES was skewed, thus not normally distributed and the non-parametric and more conservative testing method using Spearman correlations was used. For the depression questionnaire (ADS-L2), a cut-off value of ≥16 was used to separate between a low and a moderate depressive symptoms group (Radloff, 1977; Cooper et al., 2013). *T*-test group comparisons were performed for the two groups with regard to confounders and measures of body composition. To examine whether the association between age and different measures of body composition was influenced by depressive symptoms or emotional support, we conducted moderation analyses including all covariates. A comprehensive residual analysis was conducted for each model (Baddeley et al., 2005), in which we did not identify influential data points and proceeded with the regression based approach of ordinary least squares (OLS) for moderation analyses (Hayes, 2013). In order to control for family-wise errors due to multiple comparisons the results were retested with the Holm–Bonferroni method (Holm, 1979). However, because the Holm–Bonferroni method is criticized for ecological and behavioral sciences trying to detect medium to small effects due to statistical power problems, we therefore provided the cross-sectional calculated slopes for moderation analyses for better interpretation of the effects (Moran, 2003; Nakagawa, 2004). Analyses were conducted using *R* version 3.2.2 (R Core Team, 2016) and SPSS 23 (IBM Corp; Statistics). The significance level was set at α = 0.05.

## Results

Participants' characteristics are summarized in Table 1 and age and selected measures of body composition are described in Table 2. For the association between age and measures of body composition, see Table 3. All statistical significant associations between age and measures of body composition remained significant after applying the Holm–Bonferroni method to control for multiple testing. A further description of the association between age and measures of body composition as well as inter-correlations between body composition measures is provided in the Supplementary Material (Table X_A, B_).

**Table 1:** Characteristics of the sample.

	***n***	**%**
Total	269	100
Marital status		
Married or in a relationship	214	79.6
Single, divorced, or widowed	55	20.4
Ethnicity, Caucasian	261	97.0
Education		
Tertiary education	106	39.4
Post secondary non-tertiary education	57	21.2
Higher secondary school	75	27.9
Lower secondary education	29	10.7
Did not finish regular school	2	0.8
Income		
0–50,000 CHF/yr	33	12.3
50,001–100,000 CHF/yr	106	39.4
100,001–150,000 CHF/yr	90	33.5
More than 150,001 CHF/yr	40	14.8
Current smoking status		
Non-smoker	222	82.5
Occasional smoker	25	9.3
Smoker	22	8.2
Hours physical activity per week		
<1 h	9	3.3
1–3 h	90	33.5
4–6 h	109	40.5
>6 h	61	22.7
Medication intake		
No	179	66.4
Yes	90	33.6

*Data are total numbers of participants (n) and percentages (%)*.

**Table 2:** Descriptive statistics of age and measures of body composition of the sample.

	**Mean**	***SD***
Age	57.6	10.7
Weight (kg)	80.0	11.0
Height (cm)	177.4	6.5
Body mass index (BMI [kg/m^2^])	25.4	3.1
Waist girth (cm)	92.9	9.8
Hip circumference (cm)	98.0	6.8
Waist-to-hip ratio (WHR)	0.94	0.04
Fat mass (FM [%])	22.4	5.5
Fat mass (FM [kg])	18.3	6.6
Cell proportion (CP [%])	52.5	3.8
Body cell mass (BCM [kg])	32.5	4.7
Fat free mass (FFM [%])	77.6	5.5
Fat free mass (FFM [kg])	61.6	6.4
Extracellular mass (ECM [%])	25.1	5.9
Extracellular mass (ECM [kg])	29.2	3.1
Extracellular water (ECW [%])	44.4	3.7
Extracellular water (ECW [l])	19.8	2.4
Total body water (TBW [l])	44.7	5.2
ECM-BCM ratio	0.915	0.15
Water balance (WB)	7.6	56.8

*Data are means and standard deviations (SD)*.

**Table 3:** Partial correlations for age and selected measures of body composition controlled for confounders.

**Age**		**BMI**	**WHR**	**FM (%)**	**CP (%)**	**ECW (l)**	**FFM (%)**	**ECM/BCM**	**TBW (l)**	**WB**
	*r*	−0.026	0.277	0.150	−0.478	0.173	−0.150	0.448	−0.129	0.401
	*p*	0.337	0.000	0.008	0.000	0.003	0.008	0.000	0.020	0.000

*Upper values in the compartments represent correlation coefficients (r); values below represent level of significance (p)*.

### Emotional support

The BSSS comprises of two subscales emotional support (BSSS-ES) and instrumental support (BSSS-IS). Prior research investigated exclusively emotional support with regard to measures of body composition. Therefore, we provide a more detailed description of the results on emotional support. However, results for instrumental support and the main score of the BSSS—social support—are described in the Supplementary Material. Emotional support was negatively associated with age and depressive symptoms (age: *r*_*s*_ = −0.190; *p* = 0.002; ADS-L2: *r*_*s*_ = −0.196; *p* = 0.001).

**Figure 1:**
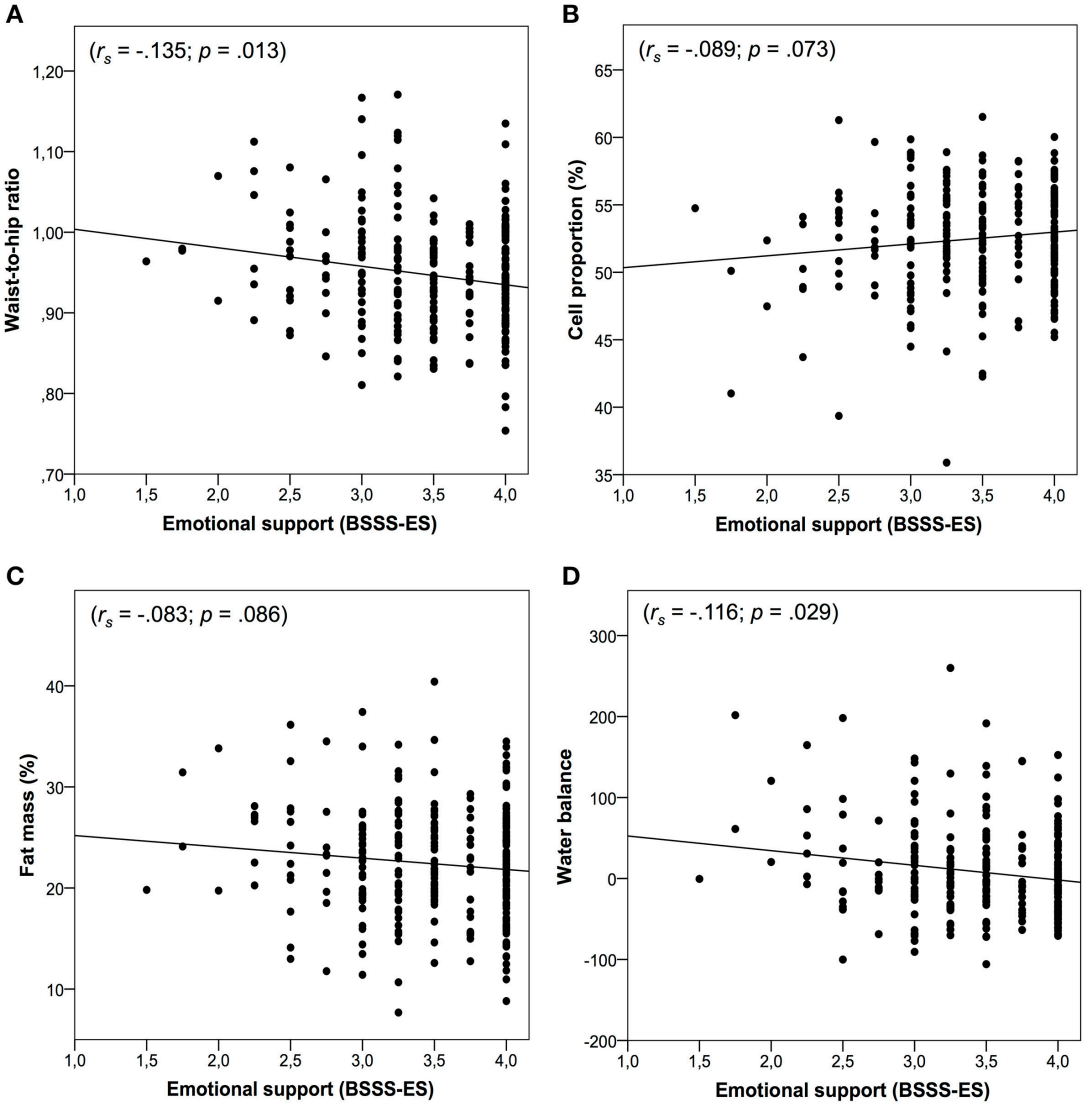
Panels **(A–D)** represent the associations between emotional support and body composition. Panel **(A)** shows the association between emotional support and waist-to-hip ratio. Panel **(B)** shows the association between emotional support and cell proportion. Panel **(C)** shows the association between emotional support and fat mass. Panel **(D)** shows the association between emotional support and water balance.

As shown in Figure 1, emotional support was negatively associated with BMI (*r*_*s*_ = −0.103; *p* = 0.045), WHR (*r*_*s*_ = −0.135; *p* = 0.013), ECMkg (*r*_*s*_ = −0.121; *p* = 0.024), ECWI (*r*_*s*_ = −0.123; *p* = 0.022), and WB (*r*_*s*_ = −0.116; *p* = 0.029). In addition, trends emerged for CP% (*r*_*s*_ = −0.089; *p* = 0.073), FM% (*r*_*s*_ = −0.083; *p* = 0.086), FMkg (*r*_*s*_ = −0.093; *p* = 0.064), ECW% (*r*_*s*_ = −0.089; *p* = 0.073), FFM% (*r*_*s*_ = −0.083; *p* = 0.086), and ECM-BCM ratio (*r*_*s*_ = −0.089; *p* = 0.073). Emotional support did not show significant associations with BCMkg (*r*_*s*_ = 0.003; *p* = 0.481), FFMkg (*r*_*s*_ = −0.059; *p* = 0.166), ECM% (*r*_*s*_ = 0.014; *p* = 0.410), or TWBl (*r*_*s*_ = −0.068; *p* = 0.133). However, after applying the Holm–Bonferroni method to control for multiple testing none of the significant associations between emotional support and measures of body composition remained significant.

**Figure 2:**
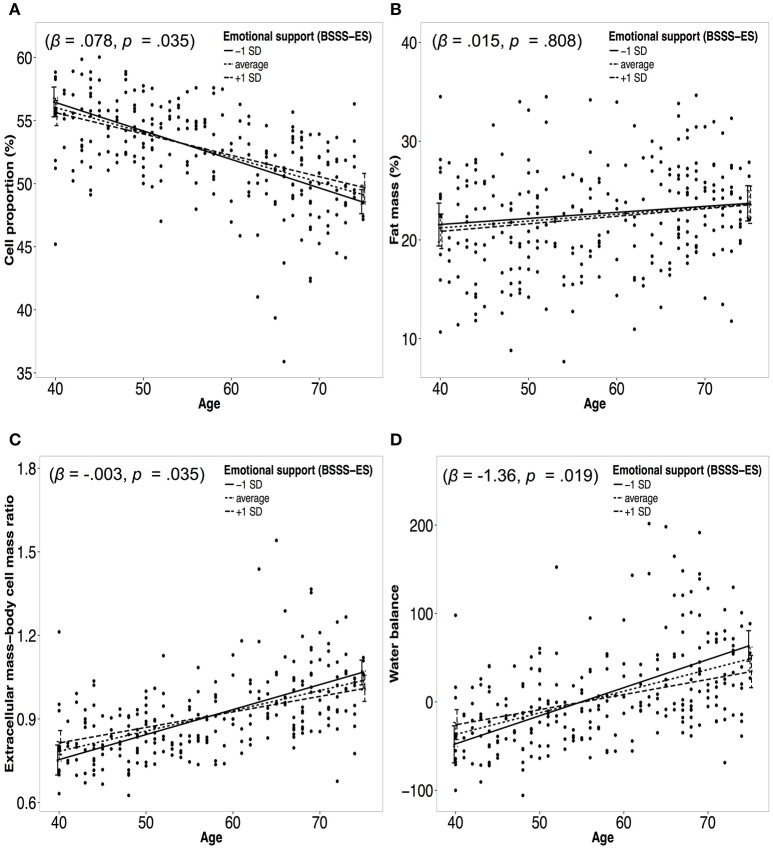
Panels **(A–D)** represent moderation plots of the relationship between age and body composition moderated by emotional support. Dotted lines represent the association between age and a measure of body composition for average emotional support. Solid/dashed lines represent the association between age and a measure of body composition for emotional support one standard deviation (*SD*) below/above the mean. Panel **(A)** represents the moderation effect of emotional support on the association between age and cell proportion. Panel **(B)** represents the moderation effect of emotional support on the association between age and fat mass. Panel **(C)** represents the moderation effect of emotional support on the association between age and extracellular mass-body cell mass ratio. Panel **(D)** represents the moderation effect of emotional support on the association between age and water balance.

Figure 2 presents moderation analyses of the relationship between age and measures of body composition moderated by emotional support and controlled for confounders. Significant effects emerged for CP% (β = 0.0785, *p* = 0.034), ECW% (β = −0.0755, *p* = 0.035), WB (β = −1.3607, *p* = 0.019), and ECM-BCM ratio (β = 0.0032; *p* = 0.035). No significant moderation effects were found for WHR (β = 0.0004, *p* = 0.572), BMI (β = 0.0234, *p* = 0.4912), FM% (β = 0.01451, *p* = 0.808), FFM% (β = −0.0145, *p* = 0.8087), and TBWl (β = −0.0208; *p* = 0.7298; see Table 4). However, after applying the Holm–Bonferroni method to control for multiple testing none of the significant moderation effects for emotional support on the association between age and measures of body composition remained significant.

**Table 4:** Moderation analyses for emotional support (BSSS-ES) and depressive symptoms (ADS-L2) on the associations between age and measures of body composition including covariates.

	**Emotional support (BSSS-ES)**					**Depressive symptoms (ADS-L2)**			
	**Estimate**	**Std. Error**	***t-*value**	**Pr (>|*t*|)**		**Estimate**	**Std. Error**	***t-*value**	**Pr (>|*t*|)**
Body mass index (BMI)									
Age:BSSS-ES	0.02342	0.03397	0.689	0.49126	Age:ADS-L2	0.04214	0.04598	0.916	0.3603
Waist-hip ratio (WHR)									
Age:BSSS-ES	0.00043	0.00076	0.566	0.5719	Age:ADS-L2	1.671e−03	1.02e-03	1.629	0.1045
Fat mass (FM %)									
Age:BSSS-ES	0.01451	0.05990	0.242	0.8087	Age:ADS-L2	0.16511	0.08054	2.050	0.0414^*^
Cell proportion (CP)									
Age:BSSS-ES	0.07850	0.03694	2.125	0.03455^*^	Age:ADS-L2	−0.11717	0.04934	−2.375	0.0087^**^
Fat free mass (FFM)									
Age:BSSS-ES	0.01451	0.05990	−0.242	0.80878	Age:ADS-L2	−0.16511	0.08054	−2.050	0.04140^*^
Extracellular mass—body cell mass ratio (ECM-BCM Ratio)									
Age:BSSS-ES	−0.00315	0.001489	−2.118	0.035140^*^	Age:ADS-L2	0.0052470	0.0019814	2.648	0.0182^*^
Total body water (TBW l)									
Age:BSSS-ES	−0.02081	0.06020	−0.346	0.729807	Age:ADS-L2	−0.008445	0.08206	−0.103	0.918
Extracellular water (ECW %)									
Age:BSSS-ES	−0.07547	0.03551	−2.125	0.03455^*^	Age:ADS-L2	0.11265	0.04744	2.375	0.0183^*^
Water balance (WB)									
Age:BSSS-ES	−1.3607	0.5785	−2.352	0.0194^*^	Age:ADS-L2	1.9488	0.7712	2.527	0.0121^*^

* *Significant moderation effect on level of significance 0.05*.

** *Significant moderation effect on level of significance 0.01*.

*Significant p < 0.05 are in bold*.

In the following, the cross-sectional calculated slopes for the relationship between age and body composition moderated by emotional support one *SD* below (−1 *SD*) and above (+1 *SD*) the mean are reported. An annual decline in CP of −0.24% was calculated for −1 *SD* in emotional support, while for +1 *SD* in emotional support an annual decline of −0.15% emerged. ECW increased 0.23 for −1 *SD* in emotional support and 0.15 for +1 *SD* in emotional support. WB increased for −1 *SD* in emotional support 3.18, while for +1 *SD* in emotional support an annual increase of 1.71 in WB was calculated. For −1 *SD* and +1 *SD* an annual increase in ECM-BCM ratio was observed of 0.0090 and of 0.0056, respectively.

### Depressive symptoms

There was no association between the amount of depressive symptoms and age (*r* = −0.065; *p* = 0.29). Two hundred twenty-five participants (83.6%) scored below the cut-off (ADS-L2 < 16) and were assigned to the low depressive symptoms group, while 44 participants (16.4%) scored at or above the cut-off (ADS-L2 ≥16) and were assigned to the moderate depressive symptoms group. The seven participants who reported using antidepressant medication scored 16 or higher on the ADS-L2. The mean ADS-L2 scores for the low and moderate depressive symptoms groups were 7.02 and 21.29, respectively. *T*-tests revealed no statistically significant differences between the two groups for the potential confounding variables with the exception of the self-reported current health condition, which was worse in the moderate depressive symptoms group.

**Figure 3:**
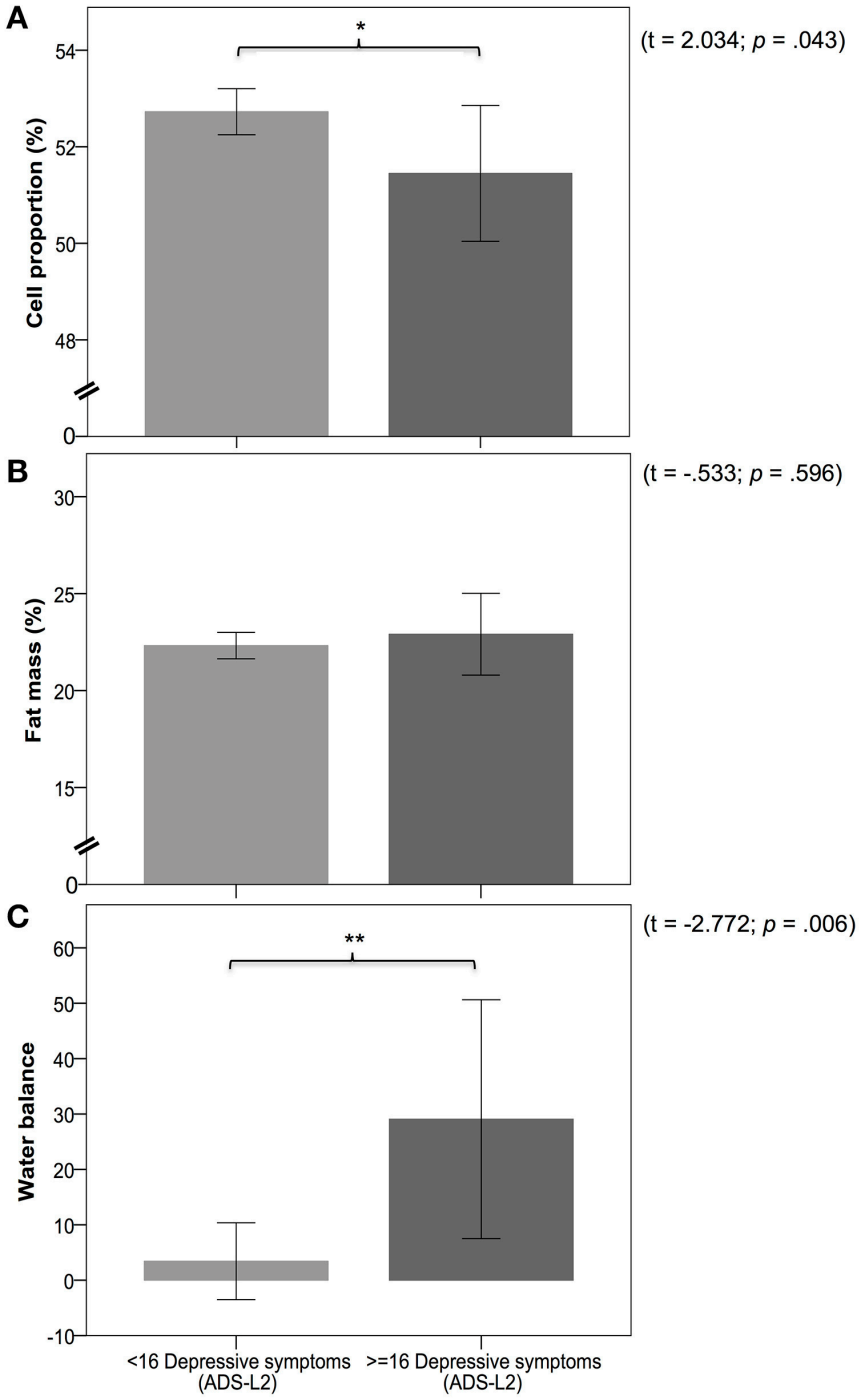
Panels **(A–C)** represent the differences in measures of body composition between the low (< 16 depressive symptoms) and moderate (≥16 depressive symptoms) depressive symptoms group. Panel **(A)** shows a comparison of cell proportion between groups. Panel **(B)** shows a comparison of fat mass between groups. Panel **(C)** shows a comparison of water balance between groups. Legend: ^*^ and ^**^ indicate significance at *p* < 0.05 and *p* < 0.01.

As shown in Figure 3, group comparisons using *T*-tests revealed significant group differences in CP% (*t* = 2.03; *p* = 0.043), ECMkg (*t* = −2.34; *p* = 0.018), ECW% (*t* = −2.03; *p* = 0.043), ECWI (*t* = −2.372; *p* = 0.018), and WB (*t* = −2.772; *p* = 0.006). *T*-tests did not reveal significant group differences between the low and the moderate depressive symptoms group for BMI (*t* = −1.84; *p* = 0.854), WHR (*t* = −1.74; *p* = 0.083), FM% (*t* = −0.533; *p* = 0.596), FMkg (*t* = −0.968; *p* = 0.338), BCMkg (*t* = 0.349; *p* = 0.727), FFM% (*t* = −0.533; *p* = 0.596), FFMkg (*t* = −0.883; *p* = 0.378), and TBWl (*t* = −1.067; *p* = 0.287). However, after applying the Holm–Bonferroni method to control for multiple testing only the difference in WB remained significant.

**Figure 4:**
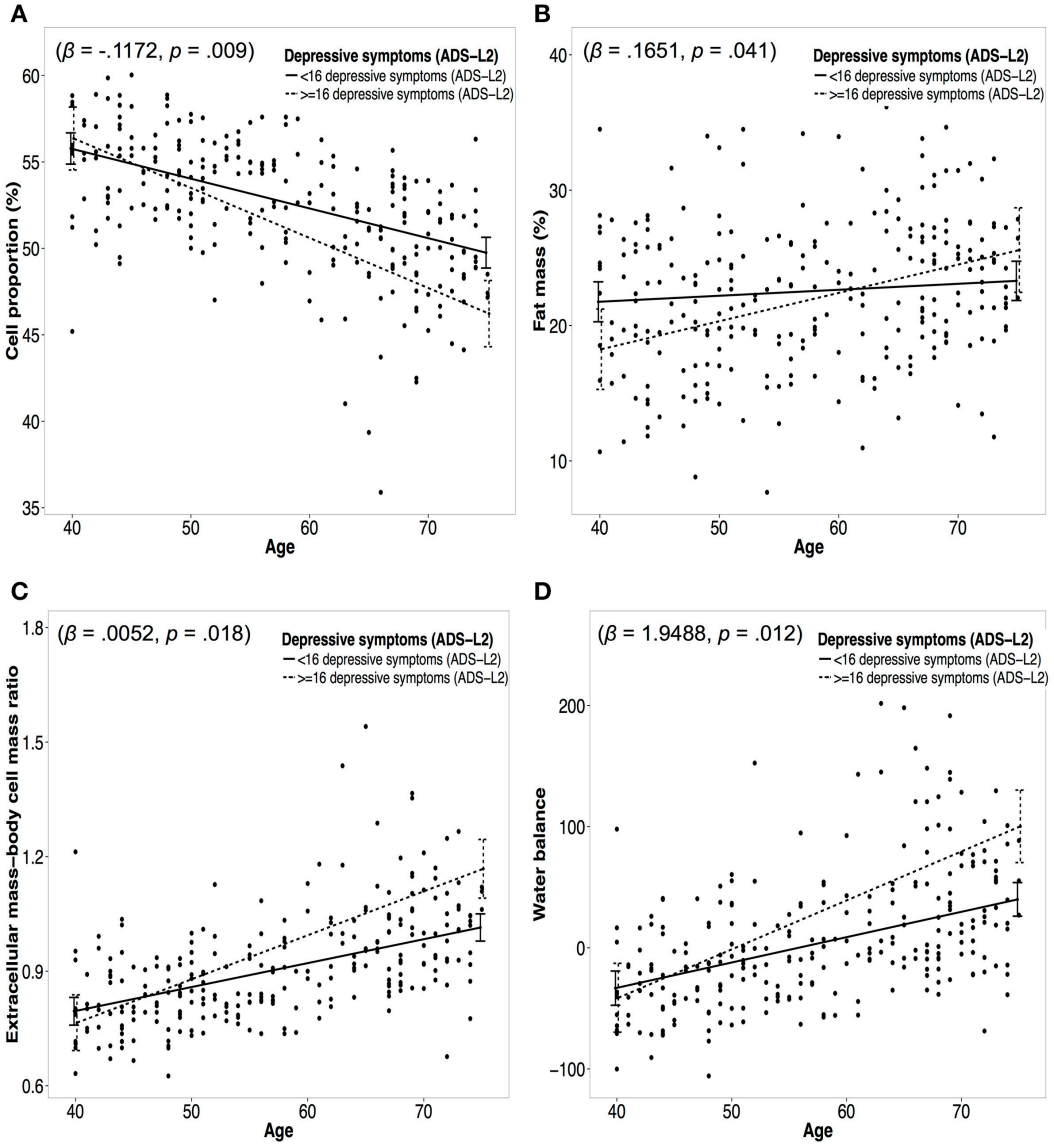
Panel **(A–D)** represent moderation plots of the relationship between age and body composition moderated by depressive symptoms. Dotted lines represent the association between age and a measure of body composition for the low depressive symptoms group (<16 depressive symptoms). Solid lines represent the association between age and a measure of body composition for the moderate depressive symptoms group (≥16 depressive symptoms). Panel **(A)** represents the moderation effect of depressive symptoms on the association between age and cell proportion. Panel **(B)** represents the moderation effect of depressive symptoms on the association between age and fat mass. Panel **(C)** represents the moderation effect of depressive symptoms on the association between age and extracellular mass-body cell mass ratio. Panel **(D)** represents the moderation effect of depressive symptoms on the association between age and water balance.

Figure 4 presents moderation analyses of the relationship between age and measures of body composition moderated by depressive symptoms and controlled for confounders. Significant group effects emerged for CP% (β = −0.1172, *p* = 0.009), FM% (β = 0.1651, *p* = 0.041), FFM% (β = −0.1651, *p* = 0.041), ECW% (β = 0.1127, *p* = 0.018), WB (β = 1.9488, *p* = 0.012), and ECM-BCM ratio (β = 0.0052; *p* = 0.018). No moderation effects were identified for BMI (β = 0.0421, *p* = 0.360), WHR (β = 1.671, *p* = 0.105), and TBWl (β = −0.0085; *p* = 0.918; see Table 4). However, after applying the Holm–Bonferroni method to control for multiple testing only the moderation effects for CP and WB remained significant.

In the following, cross-sectional calculated slopes for the two different groups (low depressive symptoms vs. moderate depressive symptoms) are reported in relation to their moderating effect between age and body composition. For the group with low depressive symptoms an annual decline in CP% of −0.17% was calculated, while for the group with moderate depressive symptoms an annual decline of −0.29% emerged. For FM% an annual increase of 0.044 for the group with low depressive symptoms emerged, whereas the group with moderate depressive symptoms increased 0.21 per year. For the low depressive symptoms group an annual decrease of −0.044% in FFM% emerged and for the moderate depressive symptoms group an annual decrease of −0.21% emerged. ECW% increased for the group with low depressive symptoms 0.17%, while the group with moderate depressive symptoms depicted an annual increase of 0.28%. WB increased for the group with low depressive symptoms 2.09, whereas it increased 4.04 for the group with moderate depressive symptoms. For the low and moderate depressive symptoms groups an annual increase in ECM-BCM ratio was observed of 0.0063 and 0.0115, respectively.

## Discussion

In this cross-sectional study comprising 269 self-reporting healthy men aged between 40 and 75 years, associations between emotional support and specific measures of body composition were observed. Emotional support had a significant moderating effect on different measures of body composition, with some moderating effects only emerging after inclusion of confounders. However, applying the Holm–Bonferroni method for multiple testing no direct or moderating effect for emotional support remained significant. Significant associations emerged between depressive symptoms and specific measures of body composition. Also after controlling for multiple testing with the Holm–Bonferroni method a significant association with WB remained indicating WB to be the most sensitive parameter to detect mood related changes in body composition. Moderating effects of depressive symptoms were observed between age and specific measures of body composition. These associations were independent of a variety of potential confounders. After controlling for multiple testing with the Holm–Bonferroni method moderation effects of depressive symptoms on the age-related alterations in CP and WB remained significant. A possible mechanism linking depressive symptoms with worse body composition has been suggested by Chuang and colleagues, who showed that chronic social defeat stress disrupts the regulation of lipid synthesis, leading to accumulation of triglycerides in the liver (Chuang et al., 2010). Furthermore, a chronic stress model increased adiposity in mice prior to any change in body weight, indicated by enlargement of adipocytes suggesting an increase in adipocyte proliferation. Chronic stress increased the hypothalamic expression of NPY linking the appetite-increasing effect of NPY with leptin resistance in this chronic psychosocial stress model (Lin et al., 2015). Therefore, our results lend further support to the literature linking psychosocial stress and worse body composition.

Interestingly, emotional support and depressive symptoms yielded effects of comparable size on the same measures of body composition, but in the opposite direction—though for emotional support not significant after correction for multiple testing. This indicates antagonistic mechanisms of emotional support and depressive symptoms with regard to age-related alterations in male body composition. To our knowledge, this is the first study to examine the associations of emotional, instrumental, and overall social support and depressive symptoms with age-related alterations in different measures of body composition in self-reporting healthy men. Our findings emphasize the importance of measuring different aspects of body composition in order to disentangle the complex patho- and salute-genetic pathway of psychosocial factors related to body composition. Furthermore, for the first time, emotional support emerged as a decelerating factor of the age-related deterioration of male body composition, and although the moderation effect did not remain significant after correction for multiple testing, emotional support should be further investigated in larger samples with more statistical power potentially yielding significant effects also after correction of multiple testing. At the same time, an intensifying effect of depressive symptoms on the age-related deterioration of body composition has been described.

Norm values for FFM and FM in a sample of 2,735 healthy men between the ages of 35–74 were similar to those found in our sample, indicating that our participants were physically healthy (Kyle et al., 2001). Compared to other studies examining the association between depressive symptoms and body composition (Pirlich, 2002; Kyle et al., 2005), this study consisted of a self-reporting healthy sample and probably has less variance in body composition than studies including participants with clinically defined syndromes. This might be the reason why no measure of adiposity revealed significant differences between the low and moderate depressive symptoms groups. However, the two groups did differ in specific aspects of body composition, such as CP%, ECW%, ECWl, and WB, indicating that these measures might be additional factors for the etiology and maintenance of depression, and could be potential treatment targets in men. Importantly, only WB remained significant after correction for multiple testing. For emotional support, direct associations with different measures of body composition such as BMI, WHR, ECMkg, or WB emerged, indicating a universal benefit of higher emotional support in terms of body composition in men. However, these results need to be interpreted with caution as after applying the Holm–Bonferroni method for correction of multiple testing these associations became non-significant. The fact that for CP% or FM% only trends emerged might be due to the sample size, as other studies included samples of more than 4,500 participants (Yu et al., 2014), while the current study included only 269 participants. A further explanation may be that the direct effect of emotional support on these measures of body composition was masked by different covariates. To understand the complex association between emotional support and body composition, it might be important to establish whether and how emotional support influences these covariates. The set of potential confounders included in the moderation analyses was very similar to the set of confounders used in a recent study investigating depressive symptoms and body composition (Yu et al., 2014). The measurement and inclusion of the appropriate covariates in the analyses is essential, and future studies should carefully add or delete covariates to the set of covariates proposed in our study or the study by Yu et al. (2014).

In this study, we did not observe an association between age and BMI, which has been described previously in large epidemiological studies (Kyle et al., 2001). This might be due to the high health status of the older individuals in this study. Compared to other studies, which included subjects with poor health, our sample did not include older individuals with a typically high BMI. Indeed, only one person aged over 50 had a BMI above 32 in the present study, while a large German epidemiological study revealed that more than 10% of men over 50 had a BMI above 33 (Hemmelmann et al., 2010). Older men with a BMI above 32 might represent a population with generally poorer health, which was intentionally excluded in this study. On the other hand participants did not show very low BMI reflecting again the good health status of participants not experiencing a lack of nutrition or suffering from diseases related low BMI. Only four men with BMIs under 20 (19.93, 19.86, 19.56, and 18.47) might represent too little variance to identify an age-related association for BMI as well as an U-shaped association between BMI and depression. Large studies reporting U-shaped associations between BMI and depressive symptoms consisted of a substantial number of underweight subjects (BMI < 18.5; Zhao et al., 2009; Noh et al., 2015). This might be the main reason, why no replication of the U-shaped association between BMI and depressive symptoms was achieved. However, also the fact that studies report that in men the U-shaped association diminishes with age might additionally explain the lack of association between BMI and depressive symptoms in this study on aging men (McCrea et al., 2011). The authors also mention that including confounders such as physical health further weakens the association between BMI and depressive symptoms indicating that the latent variable physical health might be directly associated with depressive symptoms and BMI might be a proxy for physical health.

Assessing body composition not only with BMI or WHR, but also with specific measures obtained through bioelectrical impedance analysis, has clear advantages regarding specificity and the disentangling of single contributions of adipose tissue, muscle mass or body water. However, there are also methodological or interpretational problems of different parameters of bioelectrical impedance analysis, which are described in the Supplementary Material or in Kotler et al. (1996). One additional problem is the correction for multiple testing in moderately powered studies. Bioelectrical impedance analysis provides a detailed picture of body composition by reporting numerous parameters. Assuming medium effect sizes, it is a critical question whether certain parameters should be included in the analyses or not because correction for multiple testing decreases Type 1 error (e.g., rejecting H_0_ when H_0_ is true) but also decreases the sensitivity to detect small to medium effects. We here provide data for associations without multiple testing following a more exploratory approach and subsequently retested these associations by correction for multiple testing identifying the most robust effects. The additionally provided data for the cross-sectional calculated slopes gives further information on the moderation effects of emotional support and depressive symptoms on age-related alterations in measures of body composition in healthy aging men.

A clear advantage of the present study compared to previous studies lay in the use of a validated emotional support scale. Although, previous studies reported significant associations between emotional support and measures of body composition, emotional support was only measured using a single item (Vogelzangs et al., 2007). While we found moderating effects of emotional support for the age-related deterioration of body composition when exploratory testing was applied, none of the associations for instrumental support remained significant after adjusting for confounders. This suggests that emotional support is more important than other social support measures in terms of improving body composition in aging men. On the one hand, this effect might be due to the high socioeconomic status of our sample, meaning that instrumental support was needed far less than emotional support by these men. On the other hand, however, it emphasizes the need to promote emotional support in men over 40 in order to maintain good health and delay age-related obesity or frailty. This is further underlined by the finding that emotional support significantly decreases with age (Keyes, 2002).

The amount of depressive symptoms in our sample was lower than in another large population-based sample of men with a mean age of 48 years (ADS-L2/CES-D: 9.7 vs. 10.7), indicating that our sample was healthy, with a low depressive burden (Cooper et al., 2013). In contrast, scores on the BSSS were rather high, indicating high emotional, and instrumental support received by significant others (Schulz and Schwarzer, 2003). As described above, a cut-off score of 16 on the ADS-L2 was chosen for group assignment (Radloff, 1977; Vogelzangs et al., 2007). The mean ADS-L2 scores of the low and moderate depressive symptoms groups were 7.02 and 21.03, respectively, which are similar to the values found in another large study using the same cut-off (3.8 and 20.9, respectively; Vogelzangs et al., 2008). The ADS-L2 represents the German validated version of the CES-D, and the authors of the validation study remark that a score of 16 or more does not reflect a psychiatric diagnosis of depression, but rather represents an elevated amount of depressive symptoms (Hautzinger et al., 2012). The two groups did not significantly differ in any of the confounding variables except for the self-reported health status. The phenomenon of worse self-reported health associated with depressive symptoms is well-described in the literature and was therefore expected by the authors and controlled for in the analyses (Ring, 2006).

A novelty of the present study is the description of TBW, ECW, and WB in relation to emotional support and depressive symptoms. Clear associations emerged, suggesting ECW and WB to be promising parameters for examining the relationship between body composition, psychosocial factors and aging. In line with our findings, a previous study reported higher amounts of ECW in relation to intracellular water in people with spinal cord injury, and a relation between muscle mass decrease and relative expansion of ECW (Tanaka et al., 2008). Another study reported total water intake in young healthy women to predict mood disturbances (Muñoz et al., 2015). Furthermore, the discrimination between TBW, ECW, and WB sheds light on the complex relationship of the body water-related parameters, the psychosocial parameters and aging.

Several limitations need to be considered when interpreting our results. A major limitation of this study lies in its cross-sectional design, meaning that causal inferences are not permitted. The differences in measures of body composition in relation to age are between-subject effects, and need to be further examined in relation to changes in body composition over time. We presented data only for whole-body bioelectrical impedance analysis, although there is some criticism on this method. We therefore provided data for individual segments such as right arm, left arm, trunk, right leg, and left leg in the Supplementary Material. Furthermore, the study sample is relatively small with 269 participants for the number of statistical tests performed, so that power is lacking when applying correction for multiple testing to detect small to medium sized effects. Another limitation, albeit an intentional one, is that the results only apply to self-reporting healthy men aged between 40 and 75 years. Finally, as the general socioeconomic status in Switzerland is very high compared to other countries, this sample consisted of men with high socioeconomic status.

## Conclusions

Age-related deterioration of body composition is an unavoidable reality, but its extent seems to be influenced by individual characteristics. More depressive symptoms, especially exceeding a certain threshold, substantially increased age-related deterioration of male body composition reflected by CP and WB. Furthermore, emotional support, and not instrumental support or social support in general, might play a counteracting role by decelerating age-related deterioration of male body composition. However, studies with a larger sample size, or longitudinal designs are needed to detect small to medium effects surviving correction for multiple testing. To our knowledge, this is the first study to describe a moderating effect of emotional support and depressive symptoms in parallel on the age-related deterioration of male body composition. Our results highlight the importance of taking a multidisciplinary approach in order to achieve successful aging in men, not only by focusing on exercise or nutritional and hormonal supplementation, but also by establishing and promoting psychosocial resilience as previously described (Walther and Ehlert, 2015; Walther et al., 2017a,b). We conclude that monitoring the age-related deterioration of body composition on the basis of different measures is useful to initiate timely and effective multidimensional interventions to delay age-related overweight, obesity, sarcopenia, or frailty, before they have become an irreversible reality.

## Author contributions

AW contributed to the design of the study and the data collection, analysis and interpretation of the data and wrote the first draft of the manuscript. UE contributed to the design of the study and critically revised the manuscript. NL contributed to the study design. MP contributed to the statistical analysis and the graphical representation of the data.

### Conflict of interest statement

The authors declare that the research was conducted in the absence of any commercial or financial relationships that could be construed as a potential conflict of interest.

## Abbreviations

ADS-L2: Allgemeine Depressionsskala-Langform 2
BCM: body cell mass
BMI: body mass index
BSSS: Berlin Social Support Scales
BSSS-ES: Berlin Social Support Scales-Emotional Support
BSSS-IS: Berlin Social Support Scales-Instrumental Support
CP: cell proportion
ECM: extracellular mass
ECM-BCM: extracellular mass-body cell mass ratio
ECW: extracellular water
ICW: intracellular water
FFM: fat free mass
FM: fat mass
TBW: total body water
WB: water balance
WHR: waist-to-hip ratio.
