# Supplementary material for: Emotional Support, Depressive Symptoms, and Age-Related Alterations in Male Body Composition: Cross-Sectional Findings from the Men's Health 40+ Study
Source: Front Psychol. 2017 Jun 29;8:1075. doi: 10.3389/fpsyg.2017.01075 (PMC5489702; doi:10.3389/fpsyg.2017.01075)
Supplement: Supplementary file 1 [file Table1.DOCX]

**Emotional Support, Depressive Symptoms and Age-Related Alterations in Male Body Composition: Findings From the Men’s Health 40+ Study**

Andreas Walther^1, 2^, Michel Philipp^3^, Niclà Lozza^1^ & Ulrike Ehlert^1, 2^*

Correspondence: Prof. Dr. U. Ehlert: u.ehlert@psychologie.uzh.ch

^1^Clinical Psychology and Psychotherapy, University of Zurich,

Zurich, Switzerland

^2^University Research Priority Program – Dynamics of Healthy Aging, University of Zurich,

Zurich, Switzerland

^3^Psychological Methods, Evaluation and Statistics, University of Zurich,

Zurich, Switzerland

**Supplementary Material**

| Total body water (TBW) | l | Total fluid within the body |
| --- | --- | --- |
| Extracellular water (ECW) | %  l | Sum of interstitial fluid and blood plasma (including urine) |
| Intracellular water (ICW) | %  l | Fluid within the cells of tissue |
| Water balance (WB) |  | Percentiles of the relation of extracellular water in relation to body cell mass |
| Body cell mass (BCM) | kg | All metabolically active cells sorrounded by a membrane (including muscel mass and cells of organs) and intracellular water |
| Cell proportion (CP) | % | Proportion of body cell mass in relation to fat free mass |
| Fat free mass (FFM) | kg  % | Total body mass minus fat free mass (including body cell mass, extracellular water, and bone mass) |
| Fat mass (FM) | kg  % | Total body mass minus fat free mass |
| Extracellular mass (ECM) | kg  % | Not cellular bound proportion of the fat free mass (including mainly extracellular fluid but also bone mass and connective tissue) |
| Extracellular mass – body cell mass ratio (ECM-BCM-ratio) |  | Relation of extracellular mass to body cell mass |

**Overview and description of bioelectrical impedance measures**

**Tables X_A,B_ present associations between age and measures of body composition controlled for confounders**

**Table X_A_ - extended**

**Partial correlations of measures of body composition and age controlled for confounders**^a^

|  | Age | BMI | WHR | FM (%) | FM (kg) | BCM (kg) | CP (%) | ECW (I) | ECW (%) | FFM (%) | FFM (kg) | ECM (%) | ECM (kg) | ECM-BCM ratio | TBW (I) | WB |
| --- | --- | --- | --- | --- | --- | --- | --- | --- | --- | --- | --- | --- | --- | --- | --- | --- |
| Age | 1,000 | -,026 | ,277 | ,150 | ,071 | -,362 | -,478 | ,173 | ,478 | -,150 | -,181 | ,167 | ,156 | ,448 | -,129 | ,401 |
|  | . | ,337 | ,000 | ,008 | ,130 | ,000 | ,000 | ,003 | ,000 | ,008 | ,002 | ,004 | ,006 | ,000 | ,020 | ,000 |
| BMI |  | 1,000 | ,362 | ,750 | ,862 | ,516 | ,221 | ,443 | -,221 | -,750 | ,551 | -,837 | ,375 | -,212 | ,619 | ,002 |
|  | 0 | . | ,000 | ,000 | ,000 | ,000 | ,000 | ,000 | ,000 | ,000 | ,000 | ,000 | ,000 | ,000 | ,000 | ,486 |
| WHR |  |  | 1,000 | ,423 | ,395 | ,010 | -,151 | ,227 | -,151 | -,423 | ,113 | -,295 | ,215 | ,152 | ,140 | ,209 |
|  |  |  | . | ,000 | ,000 | ,435 | ,008 | ,000 | ,008 | ,000 | ,036 | ,000 | ,000 | ,008 | ,013 | ,000 |
| FM (%) |  |  |  | 1,000 | ,935 | -,011 | -,228 | ,350 | ,228 | -1,000 | ,134 | -,781 | ,291 | ,220 | ,217 | ,317 |
|  |  |  |  | . | ,000 | ,428 | ,000 | ,000 | ,000 | ,000 | ,016 | ,000 | ,000 | ,000 | ,000 | ,000 |
| FM (kg) |  |  |  |  | 1,000 | ,265 | -,115 | ,546 | ,115 | -,935 | ,436 | -,793 | ,505 | ,108 | ,500 | ,310 |
|  |  |  |  |  | . | ,000 | ,033 | ,000 | ,033 | ,000 | ,000 | ,000 | ,000 | ,042 | ,000 | ,000 |
| BCM (kg) |  |  |  |  |  | 1,000 | ,709 | ,330 | -,709 | ,011 | ,890 | -,444 | ,365 | -,696 | ,820 | -,410 |
|  |  |  |  |  |  | . | ,000 | ,000 | ,000 | ,428 | ,000 | ,000 | ,000 | ,000 | ,000 | ,000 |
| CP (%) |  |  |  |  |  |  | 1,000 | -,394 | -1,000 | ,228 | ,314 | -,430 | -,389 | -,989 | ,236 | -.917 |
|  |  |  |  |  |  |  | . | ,000 | ,000 | ,000 | ,000 | ,000 | ,000 | ,000 | ,000 | ,000 |
| ECW (I) |  |  |  |  |  |  |  | 1,000 | ,394 | -,350 | ,706 | -,071 | ,963 | ,385 | ,797 | ,695 |
|  |  |  |  |  |  |  |  | . | ,000 | ,000 | ,000 | ,129 | ,000 | ,000 | ,000 | ,000 |
| ECW (%) |  |  |  |  |  |  |  |  | 1,000 | -,228 | -,314 | ,430 | ,389 | ,989 | -,236 | ,917 |
|  |  |  |  |  |  |  |  |  | . | ,000 | ,000 | ,000 | ,000 | ,000 | ,000 | ,000 |
| FFM (%) |  |  |  |  |  |  |  |  |  | 1,000 | -,134 | ,781 | -,291 | -,220 | -,217 | -,317 |
|  |  |  |  |  |  |  |  |  |  | . | ,016 | ,000 | ,000 | ,000 | ,000 | ,000 |
| FFM (kg) |  |  |  |  |  |  |  |  |  |  | 1,000 | -,326 | ,749 | -,309 | ,957 | ,042 |
|  |  |  |  |  |  |  |  |  |  |  | . | ,000 | ,000 | ,000 | ,000 | ,254 |
| ECM (%) |  |  |  |  |  |  |  |  |  |  |  | 1,000 | -,020 | ,431 | -,352 | ,295 |
|  |  |  |  |  |  |  |  |  |  |  |  | . | ,372 | ,000 | ,000 | ,000 |
| ECM (kg) |  |  |  |  |  |  |  |  |  |  |  |  | 1,000 | ,380 | ,763 | ,681 |
|  |  |  |  |  |  |  |  |  |  |  |  |  | . | ,000 | ,000 | ,000 |
| ECM-BCM ratio |  |  |  |  |  |  |  |  |  |  |  |  |  | 1,000 | -,234 | ,920 |
|  |  |  |  |  |  |  |  |  |  |  |  |  |  | . | ,000 | ,000 |
| TBW (I) |  |  |  |  |  |  |  |  |  |  |  |  |  |  | 1,000 | ,131 |
|  |  |  |  |  |  |  |  |  |  |  |  |  |  |  | . | ,018 |
| WB |  |  |  |  |  |  |  |  |  |  |  |  |  |  |  | 1,000 |
|  |  |  |  |  |  |  |  |  |  |  |  |  |  |  |  | . |

^a^Upper values in the compartments represent correlation coefficients and the values below represent level of significance.

**Table X_B_**

**Partial correlations of measures of body composition for specific body parts and age controlled for confounders**^a^

|  | Age | RA FM (%) | RA FM (kg) | RA FFM (kg) | LA FM (%) | LA FM (kg) | LA FFM (kg) | Trunk FM (%) | Trunk FM (kg) | Trunk FFM (kg) | RL FM (%) | RL FM (kg) | RL FFM (kg) | LL FM (%) | LL FM (kg) | LL FFM (kg) |
| --- | --- | --- | --- | --- | --- | --- | --- | --- | --- | --- | --- | --- | --- | --- | --- | --- |
| Age | 1,000 | ,152 | ,048 | -,215 | ,180 | ,073 | -,204 | ,105 | ,092 | ,008 | ,148 | -,002 | -,412 | ,185 | ,019 | -,413 |
|  | . | ,007 | ,221 | ,000 | ,002 | ,123 | ,001 | ,046 | ,071 | ,447 | ,009 | ,487 | ,000 | ,001 | ,382 | ,000 |
| RA FM (%) |  | 1,000 | ,883 | -,271 | ,937 | ,765 | -,251 | ,874 | ,832 | ,063 | ,674 | ,554 | -,067 | ,620 | ,521 | -,040 |
|  | 0 |  | ,000 | ,000 | ,000 | ,000 | ,000 | ,000 | ,000 | ,156 | ,000 | ,000 | ,141 | ,000 | ,000 | ,262 |
| RA FM (kg) |  |  | 1,000 | ,184 | ,920 | ,958 | ,199 | ,852 | ,967 | ,398 | ,843 | ,833 | ,287 | ,789 | ,806 | ,313 |
|  |  |  | . | ,002 | ,000 | ,000 | ,001 | ,000 | ,000 | ,000 | ,000 | ,000 | ,000 | ,000 | ,000 | ,000 |
| RA FFM (kg) |  |  |  | 1,000 | -,065 | ,342 | ,990 | -,072 | ,211 | ,722 | ,333 | ,538 | ,774 | ,323 | ,548 | ,775 |
|  |  |  |  | . | ,151 | ,000 | ,000 | ,125 | ,000 | ,000 | ,000 | ,000 | ,000 | ,000 | ,000 | ,000 |
| LA FM (%) |  |  |  |  | 1,000 | ,897 | -,061 | ,930 | ,938 | ,200 | ,846 | ,732 | ,037 | ,823 | ,720 | ,043 |
|  |  |  |  |  | . | ,000 | ,164 | ,000 | ,000 | ,001 | ,000 | ,000 | ,276 | ,000 | ,000 | ,245 |
| LA FM (kg) |  |  |  |  |  | 1,000 | ,349 | ,831 | ,986 | ,487 | ,924 | ,923 | ,338 | ,897 | ,917 | ,354 |
|  |  |  |  |  |  | . | ,000 | ,000 | ,000 | ,000 | ,000 | ,000 | ,000 | ,000 | ,000 | ,000 |
| LA FFM (kg) |  |  |  |  |  |  | 1,000 | -,073 | ,217 | ,741 | ,345 | ,539 | ,740 | ,325 | ,549 | ,766 |
|  |  |  |  |  |  |  | . | ,121 | ,000 | ,000 | ,000 | ,000 | ,000 | ,000 | ,000 | ,000 |
| Trunk FM (%) |  |  |  |  |  |  |  | 1,000 | ,902 | -,045 | ,834 | ,769 | ,181 | ,793 | ,746 | ,188 |
|  |  |  |  |  |  |  |  | . | ,000 | ,237 | ,000 | ,000 | ,002 | ,000 | ,000 | ,001 |
| Trunk FM (kg) |  |  |  |  |  |  |  |  | 1,000 | ,347 | ,920 | ,902 | ,281 | ,889 | ,890 | ,295 |
|  |  |  |  |  |  |  |  |  | . | ,000 | ,000 | ,000 | ,000 | ,000 | ,000 | ,000 |
| Trunk FFM (kg) |  |  |  |  |  |  |  |  |  | 1,000 | ,368 | ,422 | ,297 | ,376 | ,442 | ,320 |
|  |  |  |  |  |  |  |  |  |  | . | ,000 | ,000 | ,000 | ,000 | ,000 | ,000 |
| RL FM (%) |  |  |  |  |  |  |  |  |  |  | 1,000 | ,939 | ,269 | ,987 | ,947 | ,290 |
|  |  |  |  |  |  |  |  |  |  |  | . | ,000 | ,000 | ,000 | ,000 | ,000 |
| RL FM (kg) |  |  |  |  |  |  |  |  |  |  |  | 1,000 | ,546 | ,909 | ,995 | ,559 |
|  |  |  |  |  |  |  |  |  |  |  |  | . | ,000 | ,000 | ,000 | ,000 |
| RL FFM (kg) |  |  |  |  |  |  |  |  |  |  |  |  | 1,000 | ,199 | ,507 | ,984 |
|  |  |  |  |  |  |  |  |  |  |  |  |  | . | ,001 | ,000 | ,000 |
| LL FM (%) |  |  |  |  |  |  |  |  |  |  |  |  |  | 1,000 | ,930 | ,204 |
|  |  |  |  |  |  |  |  |  |  |  |  |  |  | . | ,000 | ,001 |
| LL FM (kg) |  |  |  |  |  |  |  |  |  |  |  |  |  |  | 1,000 | ,518 |
|  |  |  |  |  |  |  |  |  |  |  |  |  |  |  | . | ,000 |
| LL FFM (kg) |  |  |  |  |  |  |  |  |  |  |  |  |  |  |  | 1,000 |
|  |  |  |  |  |  |  |  |  |  |  |  |  |  |  |  | . |

^a^Upper values in the compartments represent correlation coefficients and the values below represent level of significance.

Abbreviations: RA = right arm, LA = left arm, RL = right leg, LL = left leg

**Tables Y_A-C_ show results for social support (BSSS) and instrumental support (BSSS-IS)**

| **Table Y_A_**  **Spearman correlations (*r_s_*) for psychosocial measures (BSSS & ADS-L2) and age** | | | | | | | | | | | |
| --- | --- | --- | --- | --- | --- | --- | --- | --- | --- | --- | --- |
| **Measure** | N | M | SD |  | 1. | 2. | 3. | 4. | 5. | 6. |  |
|  |  |  |  |  |  |  |  |  |  |  |  |
| 1. Social support (BSSS) | 269 | 2.96 | .41 |  | _ | **.767***** | **.759***** | **-.112*** | -.094^t^ | **-.135*** |  |
|  |  |  |  |  |  |  |  |  |  |  |  |
| 2. Emotional support (BSSS-ES) | 269 | 3.48 | .54 |  | **.767***** | _ | **.789***** | **-.196**** | **-.136*** | **-.190**** |  |
|  |  |  |  |  |  |  |  |  |  |  |  |
| 3. Instrumental support (BSSS-IS) | 269 | 3.44 | .54 |  | **.759***** | **.789***** | _ | **-.234***** | **-.155**** | **-.143**** |  |
|  |  |  |  |  |  |  |  |  |  |  |  |
| 4. Depressive symptoms (ADS-L2) | 269 | 9.36 | 6.9 |  | **-.112*** | **-.196**** | **-.234***** | _ | **.642***** | -.055 |  |
|  |  |  |  |  |  |  |  |  |  |  |  |
| 5. ADS-L2 Group (1=<16 vs. 2=≥16) | 269 | 1.16 | .37 |  | -.094^t^ | **-.136*** | **-.155**** | **.642***** | **_** | -.023 |  |
|  |  |  |  |  |  |  |  |  |  |  |  |
| 6. Age | 269 | 1.16 | .37 |  | **-.135*** | **-.190**** | **-.143**** | -.055 | -.023 | **_** |  |

**_____________________________________________________________________________________________________**

M = Mean; SD = standard deviation. Significant correlations in bold. *** = p < .001; ** = p < .01; * p < .05;  ^t^ = p < .1.

| **Table Y_B_**  **Selected bivariate spearman correlations (*r_s_*) for BSSS and BSSS-IS and measures of body composition** | | | | | | | | | | | | | | | | |  |
| --- | --- | --- | --- | --- | --- | --- | --- | --- | --- | --- | --- | --- | --- | --- | --- | --- | --- |
| **Measure** | BMI | WHR | CP(%) | BCM(kg) | FM(%) | FM(kg) | FFM(%) | ECW(%) | TBW(l) | WB | **ECM-BCM ratio** |  |  |  |  |  |  |
|  |  |  |  |  |  |  |  |  |  |  |  |  |  |  |  | | |
| Social support (BSSS) | -.029 | -.068^t^ | **.118*** | .089^t^ | -.040 | -.025 | .040 | **-.118*** | .018 | **-.106*** | **-.118*** |  |  |  |  | | |
| Instrumental support (BSSS-IS) | -.011 | -.024 | **.113*** | .081 | -.050 | -.043 | **.**050 | **-.113*** | .034 | **-.101*** | **-.113*** |  |  |  |  | | |

**_____________________________________________________________________________________________________**

Significant correlations in bold. *** = p < .001; ** = p < .01; * p < .05;  ^t^ = p < .1.

**Table Y_C_**

**Moderation analyses including covariates for associations of age and measures of body composition moderated by BSSS and BSSS-IS**

**Social support (BSSS)**  **Instrumental support (BSSS-IS)**

____________________________________________________________________________________________________________________

**Body mass index (BMI)**

Estimate Std. Error t value Pr(>|t|) Estimate Std. Error t value Pr(>|t|)

Age:BSSS 0.01691 0.04237 0.399 0.69012 Age:BSSS-IS -0.01270 0.03311 -0.384 0.70165

**Waist-hip ratio (WHR)**

Estimate Std. Error t value Pr(>|t|) Estimate Std. Error t value Pr(>|t|)

Age:BSSS -8.07e-05 9.469e-04 -0.085 0.93215 Age:BSSS-IS 0.0003414 0.00074 0.462 0.64477

**Fat mass (FM %)**

Estimate Std. Error t value Pr(>|t|) Estimate Std. Error t value Pr(>|t|)

Age:BSSS -0.02791 0.07459 -0.374 0.70854 Age:BSSS-IS 0.003125 0.058331 0.054 0.95731

**Cell proportion (CP)**

Estimate Std. Error t value Pr(>|t|) Estimate Std. Error t value Pr(>|t|)

Age:BSSS 0.077330 0.046092 1.678 0.09464^t^ Age:BSSS-IS 0.03722 0.03619 1.028 0.3047

**Fat free mass (FFM)**

Estimate Std. Error t value Pr(>|t|) Estimate Std. Error t value Pr(>|t|)

Age:BSSS 0.02791 0.07459 0.374 0.70854 Age:BSSS-IS -0.003125 0.058331 -0.054 0.95731

**Extracellular mass – body cell mass ratio (ECM-BCM ratio)**

Estimate Std. Error t value Pr(>|t|) Estimate Std. Error t value Pr(>|t|)

Age:BSSS -0.00312 0.00186 -1.680 0.09410^t^ Age:BSSS-IS -0.00156 0.00146 -1.071 0.2852

**Total body water (TBW l)**

Estimate Std. Error t value Pr(>|t|) Estimate Std. Error t value Pr(>|t|)

Age:BSSS -0.03786 0.07529 -0.503 0.61554 Age:BSSS-IS -0.08136 0.05866 -1.387 0.1667

**Extracellular water (ECW %)**

Estimate Std. Error t value Pr(>|t|) Estimate Std. Error t value Pr(>|t|)

Age:BSSS -0.074346 0.044313 -1.678 0.09464^t^ Age:BSSS-IS -0.03578 0.03479 -1.028 0.3047

**Water balance (WB)**

Estimate Std. Error t value Pr(>|t|) Estimate Std. Error t value Pr(>|t|)

Age:BSSS -1.50788 0.72173 -2.089 **0.03769*** Age:BSSS-IS -0.9213 0.5663 -1.627 0.1050

_________________________________________________________________________________________________________________

^*^ significant moderation effect on level of significance .05

^**^ significant moderation effect on level of significance .01

^t^ trend – below the level of significance .1

**Potential covariates and confounders (extended with references)**

Covariates included were age (1), marital status (married or in a relationship/single, separated, divorced, widowed) (2), smoking status (“non smoker”, “occasional smoker”, “1-5 cigarettes per day”, “6-10 cigarettes per day”, “more than 10 cigarettes per day”) and alcohol consumption (“never”, “seldom”, “1-2 alcoholic drinks per week”, “3-6 alcoholic drinks per week”, “1 alcoholic drink per day”, “2 or more alcoholic drinks per day”) (3), medication intake (yes/no) (4,5), drug consumption (other than alcohol or tobacco) (yes/no) (6–8), education (highest achieved education) and income (3), having had a cold or other disease during the last two weeks (yes/no) (9–11), nutrition style (from “very healthy” to “very unhealthy”) (12), self-reported increase in intake of fatty or sweet food during the last three months (“yes”/”no”) (13). The current health condition (“How would you describe your current health condition?” with the response options from “very bad” to “very good”) (14), subjective health-effort (indicated by a continuous slider: 0 = no effort at al; 100 = maximum effort) (15), and the amount of hours of sports per week (16,17) were further included as covariates.

**Methodological or interpretational problems of different parameters of the bioelectrical impedance analysis**

****The values of *R* and Xc displayed by the bioelectrical impedance analysis are based on a series circuit containing a resistor and capacitor. Therefore, are ECW (resistor) and CP (capacitor) arranged in parallel and it has been shown, that the more CP a person has the less ECW the same person will have and vice versa. ECW% and CP% measured with a bioelectrical impedance analysis rely certainly on a biological but also on a mathematical/equational dependence between each other (1,18–20). In addition, FFM is a composit of ECW as well as other body composition measures, but ECW is a very volatile measure, because it is affected for example by the time the person urinated for the last time or how much fluid a person drunk during the last 24h. Although, participants arrived for the biological examination after overnight fasting and were told to drink a maximum of one cup of water in the morning before arriving at the laboratory, no total control for water intake was possible. Why measures related to ECW might be biased through unequal water intake or urination during the 24h before examination. The schematic representation below provides an overview of associations of bioelectrical impedance analysis measures.

**References**

1. Kyle UG, Bosaeus I, De Lorenzo AD, et al. Bioelectrical impedance analysis - Part I: Review of principles and methods. *Clin Nutr*. 2004;23(5):1226-1243. doi:10.1016/j.clnu.2004.06.004.

2. Liu H, Umberson DJ. The times they are a changin’: marital status and health differentials from 1972 to 2003. *J Health Soc Behav*. 2008;49(3):239-253. doi:10.1177/002214650804900301.

3. Quan S, Jeong J-Y, Kim D-H. The Relationship between Smoking, Socioeconomic Status and Grip Strength among Community-dwelling Elderly Men in Korea: Hallym Aging Study. *Epidemiol Health*. 2013;35:e2013001. doi:10.4178/epih/e2013001.

4. Guimarães C, Pereira LRL, Almeida CAN De, Queiroz RHC. Anthropometric and Metabolic Parameters. 2006;50:4-9.

5. Roehrborn CG, Lee M, Meehan A, Waldstreicher J. Effects of finasteride on serum testosterone and body mass index in men with benign prostatic hyperplasia. *Urology*. 2003;62(5):894-899. doi:10.1016/S0090-4295(03)00661-7.

6. Okon VE, Obembe AO, Nna VU, Osim EE. Long Term Administration of Cannabis sativa Reduces Food , Water Intake and Body Weight in Mice. *Int J Sci Res*. 2014;3(3):389-392.

7. Foltin RW, Fischman MW, Byrne MF. 1988, 11, 1-14. 1988:1-14.

8. Rodondi N, Pletcher MJ, Liu K, Hulley SB, Sidney S. Marijuana Use, Diet, Body Mass Index, and Cardiovascular Risk Factors (from the CARDIA Study). *Am J Cardiol*. 2006;98(4):478-484. doi:10.1016/j.amjcard.2006.03.024.

9. Rocha R, Santana GO, Almeida N, Lyra AC. Analysis of fat and muscle mass in patients with inflammatory bowel disease during remission and active phase. *Br J Nutr*. 2009;101(5):676-679. doi:10.1017/S0007114508032224.

10. Wolfe RR. The underappreciated role of muscle in health and disease 1 Ϫ 3. 2006:475-482.

11. Franckhauser S, Elias I, Rotter Sopasakis V, et al. Overexpression of Il6 leads to hyperinsulinaemia, liver inflammation and reduced body weight in mice. *Diabetologia*. 2008;51(7):1306-1316. doi:10.1007/s00125-008-0998-8.

12. Mithal A, Bonjour JP, Boonen S, et al. Impact of nutrition on muscle mass, strength, and performance in older adults. *Osteoporos Int*. 2013;24(5):1555-1566. doi:10.1007/s00198-012-2236-y.

13. Taveras EM. Association of consumption of fried food away from home with BMI and diet quality in older children and adolescents. *Pediatrics*. 2005;116(4):518-524. doi:10.1542/peds.2004-2732.

14. Yu ZM, Parker L, Dummer TJB. Depressive symptoms, diet quality, physical activity, and body composition among populations in Nova Scotia, Canada: Report from the Atlantic partnership for tomorrow’s health. *Prev Med (Baltim)*. 2014;61:106-113. doi:10.1016/j.ypmed.2013.12.022.

15. Mccrea R, Berger Y, King M. Body mass index and common mental disorders: exploring the shape of the association and its moderation by age, gender and education. *Int J Obes*. 2011;36(10):414-421. doi:10.1038/ijo.2011.65.

16. Berkey CS, Rockett HRH, Gillman MW, Colditz GA. One-year changes in activity and in inactivity among 10-to 15-year-old boys and girls: Relationship to change in body mass index. *Pediatrics*. 2003;111(4):836-843. doi:10.1542/peds.111.4.836.

17. Hughes VA, Frontera WR, Roubenoff R, Evans WJ, Singh MAF. Longitudinal changes in body composition in older men and women : role of body weight change and physical activity 1 – 4. *Am J Clin Nutr*. 2002;76:473-481.

18. Donald P Kotler, Santiago Burastero, Jack Wang and RNP. Prediction of body cell mass , fat-free mass , water with bioelectrical impedance analysis : sex , and disease3 and total body effects of race ,. *Am J Clin Nutr*. 1996.

19. Kyle UG, Bosaeus I, De Lorenzo AD, et al. Bioelectrical impedance analysis - Part II: Utilization in clinical practice. *Clin Nutr*. 2004;23(6):1430-1453. doi:10.1016/j.clnu.2004.09.012.

20. Kyle UG, Piccoli A, Pichard C. Body composition measurements: interpretation finally made easy for clinical use. *Curr Opin Clin Nutr Metab Care*. 2003;6:387-393. doi:10.1097/01.mco.0000078988.18774.3d.
